# Capability of Cartilage Extract to *In Vitro* Differentiation of Rat Mesenchymal Stem Cells (MSCs) to Chondrocyte Lineage

**Published:** 2015

**Authors:** Setareh Talakoob, Mohammad Taghi Joghataei, Kazem Parivar, Maryam Bananej, Nima Sanadgol

**Affiliations:** 1 *Department of Biology, Faculty of Biological Sciences, North branch of Islamic Azad University of Tehran, Tehran, Iran.*; 2 *Cellular and Molecular Research Center, Iran University of Medical Sciences, Tehran, Iran.*; 3 *Department of Biology, Faculty of Basic Sciences, Sciences and Researches branch of Islamic Azad University of Tehran, Tehran, Iran.*; 4 *Department of Biology, Faculty of Sciences, University of Zabol, Zabol, Iran.*

**Keywords:** Chondrocytes, cartilage extract, mesenchymal stem cells, omentum tissue

## Abstract

The importance of mesenchymal stem cells (MSCs), as adult stem cells (ASCs) able to divide into a variety of different cells is of utmost importance for stem cell researches. In this study, the ability of cartilage extract to induce differentiation of rat derived omentum tissue MSCs (rOT-MSCs) into chondrocyte cells (CCs) was investigated. After isolation of rOT-MSCs, they were co-cultured with different concentrations of hyaline cartilage extract and chondrocyte differentiation was monitored. Expression of MSCs markers was analyzed via flow cytometry. Moreover, expression of octamer- binding transcription factor-4 (Oct-4), Wilm's tumor suppressor gene-1 (WT-1), aggrecan (AG), collagen type-II (CT-II) and collagen type-X (CT-X) was analyzed using RT-PCR on 16, 18 and 21 days. Furthermore, immunocytochemistry and western blot were performed for CT-II production. Finally, proteoglycans (PGs) were examined using toluidine blue and alcian blue staining. The phenotypic characterization revealed the positive expression of CD90, CD44 and negative expression of CD45 in rOT-MSCs. These cells also expressed mRNA of Oct-4 and WT-1 as markers of omentum tissue. Differentiated rOT-MSCs in the presence of 20 µg/ ml cartilage extract expressed AG, CT-II, CT-X, and PGs as specific markers of CCs. These observations suggest that cartilage extract is potentially able to induce differentiation of MSCs into chondrocyte lineage and may be considered as an available source for imposing tissue healing on the damaged cartilage. More investigations are needed to prove *in vivo* cartilage repair via cartilage extract or its effective factors.

Damaged articular cartilage has a particularly limited capacity for auto regeneration, because of its vascularity and low cellularity. Diseases of hyaline cartilage represent one of the major health problems especially in industrialized countries with high life expectancy (1-2). As a treatment strategy, autologous chondrocyte implantation (ACI) has been used clinically for more than 20 years. However, extensive applications are limited by intrinsic problems. Among these, cell source shortage is a key issue that needs to be resolved by exploring other resources such as stem cells (3-4). MSCs can be isolated from bone marrow, synovium, periosteum, skeletal muscle and adipose tissue (5-7). *In vitro* chondrogenesis of MSCs has been investigated with the use of growth factors and cytokines, the microenvironment, including TGF-β, BMP-6, dexamethasone and insulin (8-9). Omentum is considered as a source of ASCs and the non- fat stromal cells in the expanded omental tissue, express markers of stem cell activity, such as stromal cell derived factor 1 (SDF1-α), chemokine receptor 4 (CXCR4), and Wilms’ tumor antigen 1 (WT-1) (10). Therefore, cultured omental cells could represent a readily available source of MSCs that could be used to repair and regenerate damaged tissue. Omental stromal cells (OSCs), however, are more easily obtainable in large quantities, can be harvested from the patient’s own omentum, and able to passage in culture and differentiates into target tissues (11, 12). Oct4 is critically involved in the self- renewal of undifferentiated embryonic stem cells. As such, it is frequently used as a marker for undifferentiated cells (13). Based on the well-known healing property of the MSCs, cultured OSCs could qualify as potential stem cells from the adult for articular cartilage (14). If so, the omentum would be a convenient source of ASCs that could be used to repair and possibly regenerate damaged tissues (15, 16). Cell treatement with tissue extract or nutrient supplements is a new strategy for differentiation, with the lowest cost (17, 18). Some researchers have emphasized that local environment and resident cellular populations are the major factors determining the fate of engrafted cells (19). Also, cell extracts may prove useful for investigating the molecular mechanisms of stem cell differentiation. The cultured MSCs also express on their surface CD73, CD90, CD44 and CD105, while lacking the expression of CD11b, CD14, CD19, CD34, CD45 and CD79a surface markers (20, 21). Hyaline cartilage extract is rich in different growth factors and molecules that are effective in their proliferation and differentiation (1). In this study, we decided to monitor differentiation of isolated rOT-MSCs to chondrocytes in the presence of hyaline cartilage extract.

## Materials and methods

**Isolation, culture, expansion and storage of rOT-MSCs**

Cell isolation was performed according to Chen et al. with some modifications as described below (22). Omentum (1 mm^2^ segments) was isolated from neonatal Wistar rats (*Pasteur Institute* of Iran) aged 12 days and weighting 15-16 g through abdominal surgery, washed with phosphate- buffered saline (PBS) to remove any contamination and incubated with a solution containing 2 ml trypsin (0.25%, Gibco, UK) for 15 min at 37 °C. After incubation, the cell suspensions were centrifuged at 1500 g for 5 min at 4 °C and the remaining tissue was transferred to a new flask for the next round of digestion. The procedure was repeated for 15, 20 and 40 min. After centrifugation, the cell pellet was washed with Dulbecco’s Modiﬁed Eagle’s Medium (DMEM, Gibco, UK) containing 10% fetal bovine serum (FBS, Gibco, UK) and 1% penicillin/ streptomycin (Pen/ Str, Sigma, USA), resuspended in the same medium and plated in a 25 cm^2 ^cell culture flask (Jet/ Biofil, Italy). Non- adherent cells were removed 3 days later by two brief washing with medium. The dishes were incubated in a humidified atmosphere of 95% air and 5% CO_2 _at 37 °C, and the medium was changed every three days. When cultures reached the optimum confluence, cells were lifted by incubation with PBS containing trypsin (0.25%) and 0.02% EDTA at 37 °C. Detached cells were suspended, washed in DMEM, centrifuged at 1500 g for 5 min and seeded on three new 25 cm^2^ cell culture flasks (ratio 1:3). An equal volume of stromal medium was added to inactivate the trypsin and the suspension was transferred to a conical centrifuge tube. After centrifuging at 1500 g for 5 min, the cell pellet was resuspended in 1 to 2 ml of cryopreservation medium (the same formulation as that used to propagate the cells with 20% FBS, 10% dimethyl sulfoxide (DMSO, Sigma, USA)) and transferred into a liquid nitrogen container for long- term storage (23).

**Cartilage extract preparation and treatment**

For the preparation of cartilage extract, cartilages from sternum area of 12 days rats were isolated, weighted and 6 gr of cartilage tissue was washed with sterile PBS several times to remove any contamination. Cartilage tissues were divided into smaller segments and were supplemented with 1 ml trypsin (0.25%) in PBS and incubated (15 minutes at 37 °C). Samples were washed with 1 ml PBS and centrifuged at 1000 g for 5 minutes. Supernatants were resuspended in sterile DMEM and then were placed in a glass homogenizing tube at low speed for 20- 30 sec on wet ice (Jencons, USA). The homogenate was transferred to centrifuge tubes and centrifuged at 15000 rpm for 20 min at 4 °C. Supernatants were removed with a sterile syringe (pore size 0.45 µm) and stored in aliquots at -20 °C until use (24). rOT-MSCs were plated at a number of 5× 10^4^ cells/ cm^2^ in 25 cm^2 ^plastic culture flasks without specific extracellular matrix- coated treatment and cultured in expansion medium at 37 °C, 5% CO_2_. Undifferentiated cells were harvested after 3 days of culture at 70% confluence. For differentiation assay, when OSCs reached 70% conﬂuence, they were treated with different concentrations (10, 20, 40 and 80 µg/ ml) of cartilage extract with 2%, 5%, 10% and 15% of FBS for 21 days respectively. For effective differentiation, the level of FBS gradually increased at concentrations 30 and 60 µg/ ml. The same medium without cartilage extract and 10% FBS was used as control during the differentiation. Medium changes were carried out every 4 days until day 21.

**Flow cytometry**

Cells were plated at a density of 200 cells/ cm2 on fibronectin- coated culture dishes and harvested at about 50% confluence. 10^5^- 10^6^ cells were incubated with FITC- or PE- conjugated anti- CD90, anti- CD45 and anti- CD44 for 30 min at 4 °C in PBS. The primary and secondary antibodies concentrations used were as follows: rabbit anti-CD_90_, rabbit anti- CD_45_, rabbit anti- CD_44_ dilution 1:200 and goat anti- rabbit IgG- FITC dilution 1:4 (Abcam, UK). Isotype- matched irrelevant polyclonal antibodies were used as negative controls. For cell surface staining, cells were incubated in darkness for 30 min at 4 °C in PBS supplemented with bovine serum albumin (BSA, Sigma, USA). After washing, the cells were resuspended in PBS and fluorescent staining was measured using a flow cytometer (Beckton Dickinson, USA) and the results were analyzed with the Win MDI 2.8 software (Scripps Institute, La Jolla, CA, USA).

**Reverse Transcription- Polymerase Chain Rea-ction (RT-PCR)**

RT-PCR analysis was performed for specific genes on days 16, 18 and 21 according to standard protocol with some modifications (25). Total RNA was isolated from cell lysates using TriPure isolating reagent as described by the manufacturer (Ambion, USA). Extracted RNA was solved in diethylpyrocarbonate (DEPC) treated water and digested with RNase- free DNase I (Fermentas, USA) to remove contaminating genomic DNA. DNase I was dissolved in 10X reaction buffer with MgCl_2_ and 1 µg of DNase I was added per 1 µl of RNA and incubated for 30 min at 37 ºC. DNase I activity was arrested following addition of 1 µl of 25 mM EDTA and incubation at 65 ºC for 10 min. The RT-PCR procedure was carried out in one step using 1 µg of total tissue RNA and random hexamers as primers using the RT-PCR system (12, 13, 26). The system uses AMV Reverse Transcriptase (5 U/ µl) for first strand synthesis and Taq DNA polymerase for second strand cDNA synthesis and amplification. cDNA was synthesized using BioRTcDNA first strand synthesis kit (Hangzhou Bioer technology, China) and semi- quantitative RT-PCR was carried out with 2 µl cDNA, 250 nM each of forward and reverse primers (Table 1) and 1 U Taq DNA polymerase in a final volume of 20 µl. Each cycle consisted of 2 min at 94 °C, 15 s at 58 °C, 30s at 72 °C (35 cycles) and finally 10 min at 72 °C (Super Cycler Trinity, Kyratec, Africa). Amplified DNA fragments were electrophoresed on 1.7% agarose gel, stained with Biosafe and photographed on a UV transilluminator (UVIDOC, UK).

**Immunocytochemistry assay (ICC)**

Chondrocytes markers in cells were monitored by ICC staining at days 16, 18 and 21. After 16, 18 and 21 days of cell culture under chondrocyte- conditioned medium (cartilage extract), tissue like pellets were embedded with paraffin. Sectioned (thickness of 5 μm) tissues were rehydrated and pre- treated with papain solution for 15 min. Subsequently, the sections were washed with PBS and then permeablized with blocking buffer containing 10% normal donkey serum (Zsbio, China) and 0.3% (v/v) Triton X-100 for 45 min at room temperature to block nonspeciﬁc antibody binding. Corresponding primary antibodies including the sheep anti- rat collagen type II was then added and incubated overnight at 4 °C. The cells were subsequently washed three times with PBS and incubated with a second fluorescence- labeled antibody containing goat anti- rat collagen type II FITC- conjugated IgG at room temperature, for 1 h in darkness. After washing with PBS, the cells were incubated with 1 µg/ ml 4, 6-diamidino-2-phenylindole (DAPI, dilution 1:1000; Roche, Germany) for 60 min at room temperature with the purpose of nuclear staining. Sections were mounted using mounting medium containing iodide propidium (IP) and observed under a fluorescence microscope (Nikon TE-2000, Japan) (24). To evaluate the number of immunostained cells, photographs of 10 random fields (×100) per slide were analyzed by NIH Image software (National Institutes of Health; Bethesda, MD). Negative control was performed avoiding primary antibody.

**Table 1:** Primers used for reverse transcription polymerase chain reaction of specific gene expression

**Accession no**	**Name**	**Sequence**	**Product size (bp)**
NM_007424.2	Aggrecan	F: 5'-CAAGAATCAAGTGGAGCCGTGTT-3' R: 5'-TCAAAGTCCAGTGTGTAGCGTGT-3'	295
NM_012929.1	Collagen type II	F: 5'-AGCAGCAAGAGCAAGGAGAAG-3' R: 5'-AACAATGGGAAGGCGTGAGG-3'	249
XM_008773018.1	Collagen type X	F: 5'-CAGCAGCACTATGACCCAAG-3' R: 5'-GCCGTTCGATTCCGCATT-3'	378
NM_031534	WT-1	F: 5'-TGAGAAACCATACCAGTGTGAC-3' R: 5'-GTAGGTGAGAGGGAGGAATTTC-3'	396
NM_001009178	Oct-4	F: 5'-GGAGATATGCAAATCGGAGACC-3' R: 5'-CGAGTAGAGTGTGGTGAAATGG-3'	352
NM_031144	β-actin	F: 5'-TCATGAAGTGTGACGTTGACATCCGT-3' R: 5'-CCTAGAAGCATTTGGGGTGCACGATG-3'	285

**Fig. 1:**
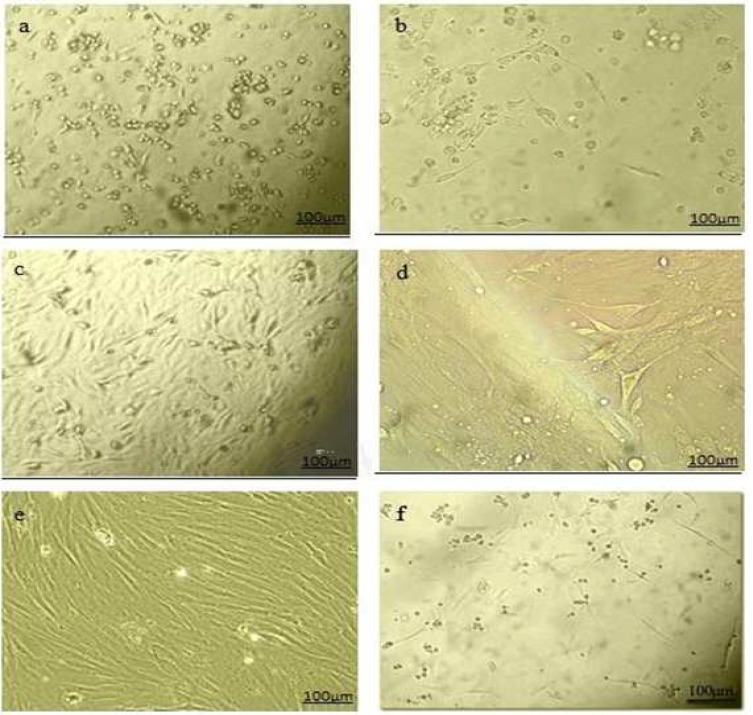
Cell morphology of cultured rOT-MSCs. The initial adherent cells appeared as separate colonies after 19 h (a), and 2 days (b). More confluent omental stromal cells were observed after 5 days (c), and monolayer cultured MSCs after one week (d). Spindle- shaped morpho-logy of cells appeared after new consecutive passages (e), and re-attachement of cells to the culture flasks following freeze- thawing (f).

**Western blotting analysis**

Induction of chondrocyte phenotypes in cells was monitored by western blotting on days 18 and 21. Western blot analysis was performed as described previously (27). Cell lysates of proteins from different culture stages were subjected to 0.1% SDS- 7.5% PAGE, and the separated proteins were transferred into a nitrocellulose membrane (Amersham) and membranes were blocked for 1.5 h using a western blocker solution (Sigma, USA) before being incubated with an antibody against collagen type II. The proteins were detected on immunoblots using primary antibody (sheep anti- rat collagen type II antibody) diluted with 0.05% Triton X-100 in tris- buffered saline containing 1% gelatin, followed by HRP- conjugated secondary antibody (HRP- labelled goat anti- rat collagen type II IgG). Immunoreactive bands were developed by ECL (Amersham) and blots were exposed to medical X-ray film (Kodak, Rochester, NY,USA). The specific bands were detected by using the enhanced chemiluminescence system (Pharmacia, Piscataway, NJ).

**Histological staining**

Induction of chondrocyte phenotypes in cells was monitored by toluidine blue and, alcian blue stains (MERCK, Germany) for the detection of proteoglycans (PGs) in the extracellular matrix (ECM) at days 16 and 21. In the chondrocyte differentiation group (6 µl cartilage extract with 2% FBS), the medium was removed from the ﬂasks, tissue like pellets were rinsed with PBS three times and ﬁxed with 10% formalin for 20 min at room temperature, dehydrated through a graded series of ethanol, embedded in paraffin, and sectioned at a thickness of 5 μm. For histological analysis, sections were de-paraffinized, rehydrated and stained with alcian and toluidine blue. Sections were incubated in 1% toluidine blue for 30 sec, and 1% alcian blue for 20 min at room temperature, then rapidly washed in water. The sections were examined and photographed with a photonic microscope (22).

**Viability assay**

Cell viability was determined by trypan blue exclusion assay. The percentage of cells excluding trypan blue was taken as a measure of cell viability in different concentrations (10, 20, 40 and 80 µg/ ml) of cartilage extracts.

**Fig. 2:**
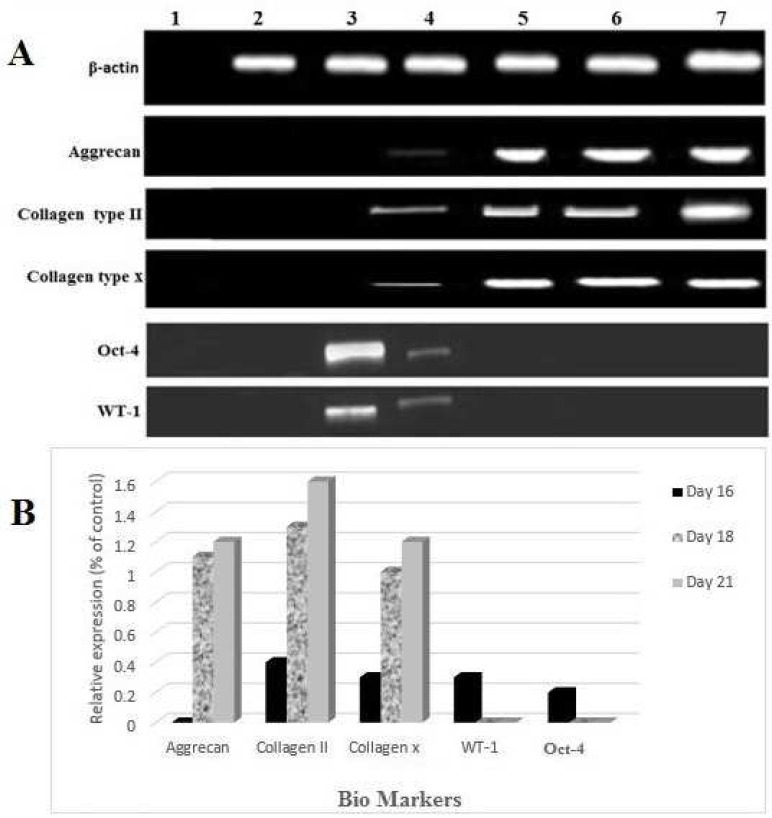
Reverse transcription polymerase chain reaction analysis of specific embryonic (Oct-4) and adult stem cells markers (WT-1) of rOT-MSCs and chondrocyte cell marker gene expression (AG, CT-II and CT-X) in differentiated MSCs (A) and its relative expression to internal control, β-actin (B). H_2_O negative control for PCR (Lane 1), breast cancer cell line SKbr3 as negative control for AG, CT-II and CT-X (Lane 2), untreated rOT-MSCs (Lane 3), 16 days differentiated cells ( Lane 4), 18 days differentiated cells (Lane 5), 21 days differentiated cells (Lane 6), chondrocyte cell line as positive control (Lane 7).

**Statistical analyzes**

The data presented were means of triplicate determinations. One- way ANOVA test was used for statistical analysis of more than one group of samples. For all other data, unpaired t-test was used. A p-value of, 0.05 was considered statisti-cally significant.

## Results

**rOT-MSCs culture and characterization**

The initial adherent spindle- shaped rOT-MSCs appeared as separate colonies at the bottom of culture ﬂasks within 19 h (Fig. 1a). Cells in 2- 5 days culture became more confluent (Fig. 1b and c), and reached 75- 80% of confluence within one week (Fig. 1d). Morphologically verified cells were lifted and cultured in another 25 cm^2^ flasks (1:3) until confluence was achieved. Cell population appeared to be more homogeneously formed of spindle-shaped cells (Fig. 1e) and following a freeze-thaw stage these cells reattached to the culture flasks with slow growth rate (Fig. 1f). Molecular analysis (RT-PCR) confirmed that the isolated cells express omental (Oct-4 and WT-1) markers (Fig. 2). 

**Fig. 3:**
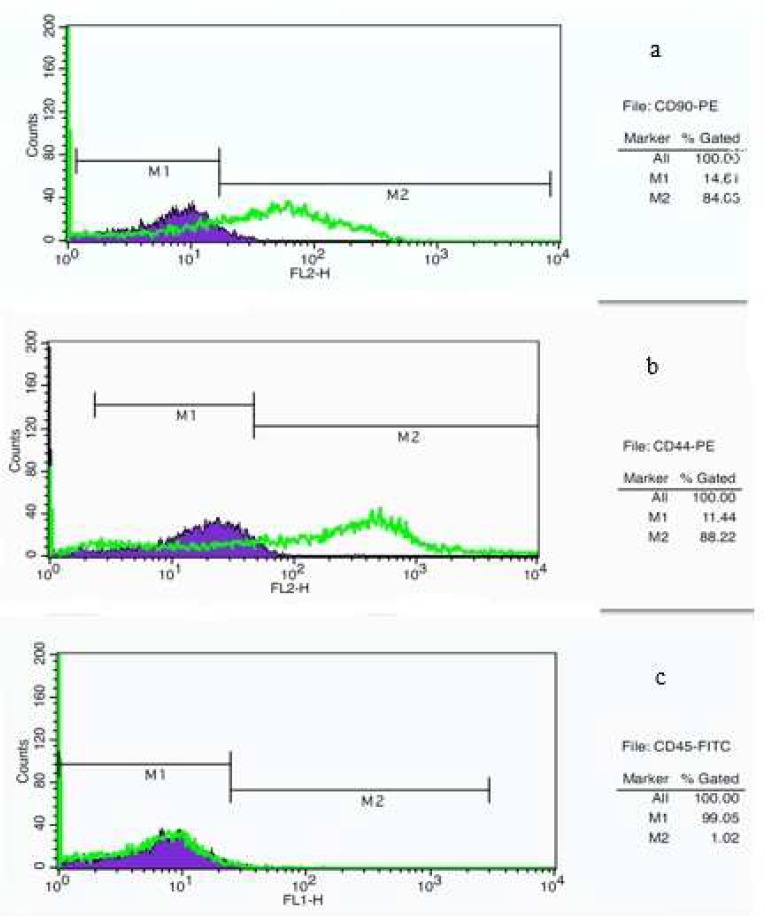
Flow cytometry analysis of cell surface markers on rOT-MSCs after one week culture. Cells were positive for CD90-PE (a), CD44-PE (b), and negative for CD45-FITC (c). M_1_: isotype control, M_2_: CD90, CD44 and CD45 antibody

Moreover, expression of cell surface mesenchymal markers (CD90 and DC44) was confirmed via flow cytometry assay (Fig. 3). Cells were let to attach and adapt with new environment during first days and then flow cytometry analysis was performed after one week treatment (75- 80% of confluence). Flow cytometry analysis revealed that the rOT-MSCs were positive for CD90 (84.05%), CD44 (88.22%) and relatively negative for CD45 (1.02%) (Fig. 3).

**Characterization of chondrocyte-like cells**

**Fig. 4:**
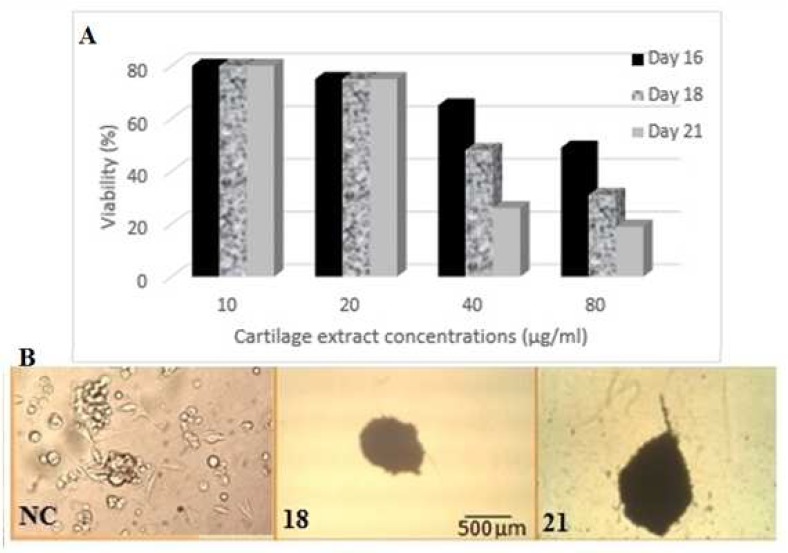
Viability assay of rOT-MSCs after 16, 18 and 21 days treatment with different concentrations of cartilage extract (A). Cell morphology of differentiated rOT-MSCs after 20 µg/ ml cartilage extract treatment were observed with an Olympus phase contrast microscope (B). rOT-MSCs as control cells with spindle-shaped morphology (NC), cells after 18 days (18), and 21 days treatement (21)

A large number of cells died from high concentrations of blocking enzymes present in cartilage extract at 40 and 80 µg/ ml concentrations (Fig. 4). The structure of chondrocyte- like cells vanished gradually in dose dependent manner and in the end stage, the cells were aged and died (Fig. 4). Observations showed that the effective concen-tration for chondrocyte differentiation was 20 µg/ ml (Fig. 4). Under 20 µg/ ml fresh cartilage extract treatment, rOT-MSCs gradually progressed toward the morphology of chondrocyte (polygonal and round) in a time- dependent manner. The morphology of the cells was observed under light microscopy (increase in the pellet size) and compared with rOT-MSCs as control (Fig. 4). The morphology of chondrocyte- like cells were observed as early as 18 days after culturing under cartilage extract and further matured by day 21 in the presence of 2% FBS (Fig. 4). Differentiation of cells to the chondrocyte phenotype was confirmed with expression of aggrecan (AG), collagen type- II (CT-II) and collagen type- X (CT-X) (Fig. 2). However, CT-II, an early chondrocyte marker, was up- regulated on day 21, suggesting that some of the cells may still be in early stages of chondrocyte lineage. In addition, the high level of CT-II expression on day 21 might be due to the proliferation of chondrocyte progenitors.

**Fig. 5:**
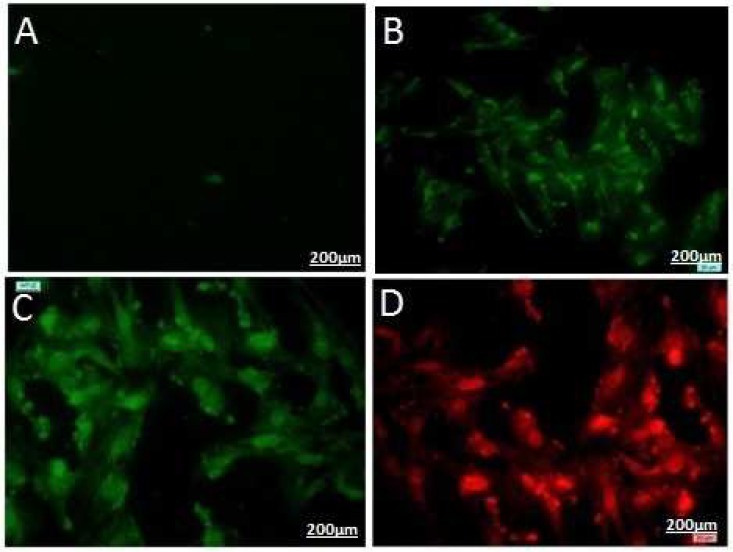
ICC staining of CT-II in differentiated cells from rOT-MSCs after 20 µg/ ml cartilage extract treatment. 16, 18, 21, 21 days differentiated cells (a, b, c, d, respectively).

**Immunocytochemical analysis of collagen type- II (CT-II)**

The presence of CT-II is a prominent feature of mature chondrocyte, as cartilage is the predominant site for the synthesis of CT-II protein. The ICC results showed that the cells expressed CT-II on day 16, 18 and 21. The percentage of CT-II positive cells was 20, 55 and 90% in the differentiated cells on 16, 18 and 21 days, respectively (Fig. 5). So, immunocytochemical staining showed significantly greater levels of CT-II after 18 days differentiation as compared with the control (P< 0.05).

**Western blotting analysis of collagen type- II (CT-II)**

**Fig. 6:**
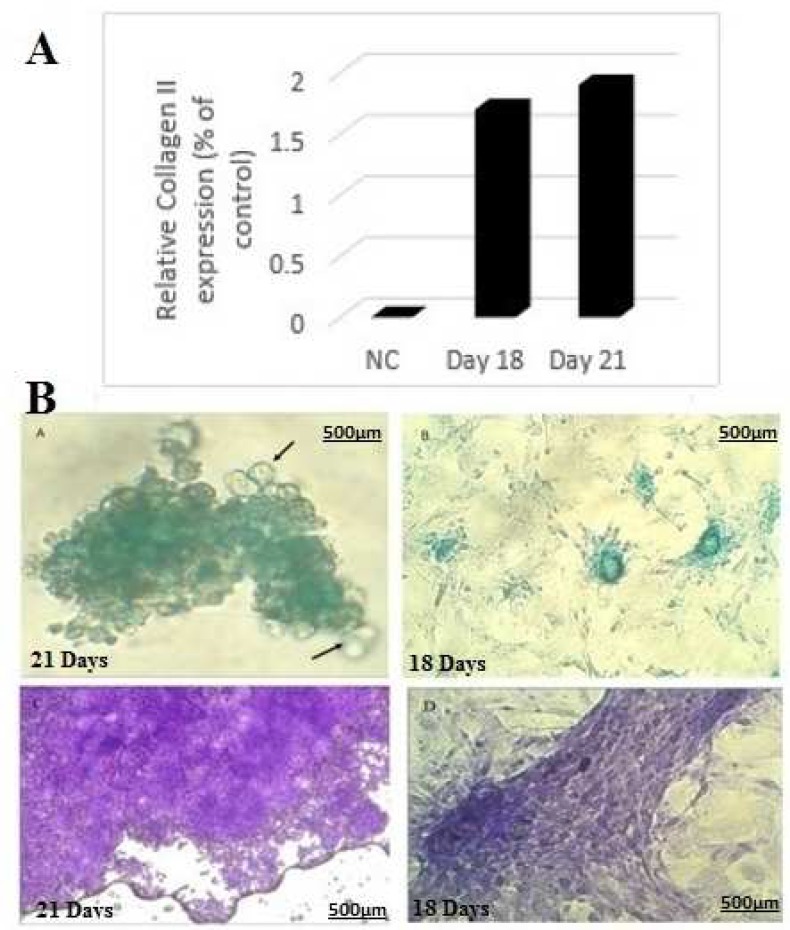
Protein expression of CT-II was confirmed by western blot analysis after treatment of rOT-MSCs with 20 µg/ ml cartilage extract. Undifferentiated rOT-MSCs as negative control (NC), 18 days differentiated cells and 21 days differentiated cells (A). Alcian blue staining for proteoglycans (PGs) production in the differentiation process of rOT-MSCs in the presence of 20 µg/ ml cartilage extract, transparent cartilage cells with spherical *shape* and green intracellular matrix (arrows) after 18 and 21 days (Ba, Bb). Toluidine blue for PGs production in the differentiation process of rOT-MSCs in presence of 20 µg/ ml cartilage extract, cartilage cells with semi-*lacona shape*, opaque nucleus and violet intra cellular matrix after 18 and 21 days (Bc, Bd

To further confirm efficient chondrocyte induction, we checked the protein expression of CT-II by western blot analysis. SDS-PAGE showed the presence of a prominent band of CT-II in the media of differentiated cells at days 18 and 21, whereas a small amount of CT-II was present in the media of undifferentiated (control) cells (Fig. 6A). The rate of CT-II secretion by the differentiated cells was 75 to 95 times higher than undifferentiated or control cells (Fig. 6A).

**Toluidine blue and alcian blue staining for proteoglycans (PGs)**

Proteoglycan production was studied for examining whether differentiated chondrocyte from omentum MSCs were functionally competent or not. After 18 and 21 days differentiation culture, induced omentum MSCs were analyzed for their proteoglycans- storage ability by toluidine blue and alcian blue staining. The majority (50% to 60%) of rOT-MSC- derived chondrocytes were strongly positive for proteoglycans at days 18 and 21 (Fig. 6B). The positive rates were 60% to 70%, while undifferentiated MSCs were negative (Fig. 6B).

## Discussion

Cartilage tissue damages are a rather worldwide problem of many people and are produced by either trauma or age dependent degenerative diseases (28). MSCs could be considered as an appropriate source for cell- based treatment of cartilage defects owing to their capacity in undergoing extensive self- renewal proliferation as well as the potential of giving rise to chondrocyte cell lineage (29). The omentum has long been known to have the power to heal injured organs once it has adhered to the damaged site, either naturally or deliberately by surgery (10). It has been previously shown that the omentum, especially after its activation by injury, becomes a reservoir of stromal cells that express stem cell markers and growth factors (16, 30). At present, proposed therapies involving the use of embryonic cells pose ethical questions and carry the risk of uncontrollable growth and tumor induction (31). By contrast, adult cells with stem cell properties are safer and have greater practical use, having been employed for over 50 years to replace bone marrow in leukemia and non- hematological diseases without causing malignant transformation (12). Adult cells with stem cell properties have previously been identified in the bone marrow mesenchyme, skin, hair, dental pulp, kidney, and even peripheral blood (32). Omental stromal cells, however, are more easily obtainable in large quantities and can be harvested from the patient’s own omentum, thus obviating the need for immunosuppressive therapy. Moreover, these cells can be passaged in culture without loss of pluripotent markers (Oct-4, Nanog) and they also can be frozen in large numbers for long- term use (33). Based on the well- known healing property of the omentum, stem cell markers characterization, secretion of high amounts of VEGF, and the ability to recognize injured sites, cultured omental stromal cells could qualify as potential stem cells from the adult. If so, the omentum would be a convenient source of adult stem cells and could be used to repair and possibly regenerate damaged tissues. In this study omental stromal cells adhered to plastic, were mesenchymal (CD90^+^, CD45^-^ and CD44^+^) and not hematopoietic in morphology and phenotype. They also expressed markers of adult (WT-1) as well as embryonic stem cells (Oct-4). Adhesion of cells to ECM compounds pre- coated dish is crucial for cell differentiation and maintenance of mesothelium cells in culture (12). Although soluble factors such as FGFs, BMPs and Wnts have been well studied for their role in regulating stem cell behavior, the effect of cell- matrix interaction in stem cell development is poorly understood. Our results showed that omental stromal cells could successfully grow in dish without ECM coating in the presence of serum and 10% FBS. The most commonly used growth factors and cytokines in chondrogenesis are TGF-β1, TGF-β3, BMP-6, BMP-2 and BMP-9 (34-36). In this research, for the first time, we cultured the omental stromal cells in plastic culture flasks without treatment with matrigel or fibronectin with relatively good differentiation capacity. In contrast, scientists released data that support the possibility that the fibronectin matrix has an instructive role in directing cells through the condensation, proliferation and/ or differentiation stages of cartilage formation (37). Nevertheless, these omental cells, that share all the other properties with the so- called stem cells, hold the highest differentiation potential, as also shown by other groups (12, 38). Furthermore, Chen et al. showed that adhesiveness to various ECM and non- biological substrates determines the differentiation of stem cell in such a way that efficient cell- cell aggregation, together with less efficient cell attachment and spreading, results in more efficient cell differentiation (39). New technologies have offered insights into how stem cells sense signals from the ECM and how they respond to these signals at the molecular level, which ultimately regulate their fate (40). These are the first findings to illustrate the mechanism of rOT-MSCs differentiation into chondrocyte cells using hyaline cartilage extract. This study indicates that the extract of neonatal rat hyaline cartilage could effectively differentiate omental stromal cells into chondrocyte-like cells via expression of specific markers such as AG, CT-II, and CT-X. These yield differentiated cells could provide a potential source of chondrocyte for drug screening *in vitro* and a valuable reference for cell therapy for cartilage tissue damages. Adult stem cells comprise the first line repair mechanism, called into action by normal wear and tear of the body as well as by any serious damage or attack caused by disease or infection (41). Nevertheless, our work contributes to the knowledge needed for the development of clinical stem cell treatment for cartilage failure. Our results also shed light on the underlying mechanism of cartilage regeneration.
